# Effects of electroconvulsive therapy on functional brain networks in patients with schizophrenia

**DOI:** 10.1186/s12888-023-05408-1

**Published:** 2024-01-08

**Authors:** Yibo Geng, Hongxing Zhang, Zhao Dong, Haisan Zhang

**Affiliations:** 1 Department of Magnetic Resonance Imaging, The Second Affiliated Hospital, Xinxiang Medical University, Henan, China; 2 Mental Hospital, Xinxiang Key Laboratory of Multimodal Brain Imaging, Xinxiang Mental Image Engineering Technology Research Center, Xinxiang, 453002 China; 3 Department of Psychology, Xinxiang Medical University, Xinxiang, 453003 China

**Keywords:** Schizophrenia, Electroconvulsive therapy, Functional network, Graph theory

## Abstract

**Background:**

Schizophrenia is a kind of intractable brain disorder. Electroconvulsive therapy (ECT) has been used to rapidly improve the clinical symptoms of patients with schizophrenia, but the effect of ECT on topological attributes of brain functional network in patients with schizophrenia has not been clear. The purpose of this study was to investigate the brain functional network mechanism of ECT against schizophrenia.

**Methods:**

Thirty-one patients with schizophrenia and fifty healthy controls matching age, gender, and years of education were included. All participants underwent general data collection and magnetic resonance imaging scanning before ECT, and clinical symptoms were assessed using the Positive And Negative Syndrome Scale (PANSS). MRI and clinical symptoms were collected again after the first and eighth ECT application. The functional brain network was constructed on the basis of magnetic resonance imaging, and the global and node topological properties were analyzed. Repeated measure variance analysis was used to explore the changes of the topological attribute values and clinical symptom scores before and after ECT, and Bonferroni post hoc analysis was performed. The independent sample t-test was used to compare the differences in the topological attribute values between patients and healthy controls at three time points before and after ECT. Partial correlation analysis was performed for topological attribute values and clinical symptom scores of abnormal brain regions in the patient groups and their changes during ECT. A general linear regression model was used to predict the outcome after the final eighth ECT using the patient's response to the first ECT.

**Results:**

(1) One ECT can restore the gamma(γ), lamuda(λ), sigma(σ), nodal global efficiency (Ne) of right insular gyrus ventral agranular insula (INS_R_vIa) and nodal local efficiency (NLe) of bilateral fusiform gyrus medioventral area37 (FuG_A37mv). Eight ECT can also restore the NLe of cortex rostral lingual gyrus (MVOcC _R_rLinG). Eight ECT did not improve the Ne of right superior parietal lobule rostral area 7 (SPL_R_A7r) and NLe of left superior frontal gyrus medial area 6 (SFG_L_A6m). (2) Even after only the first use of ECT, total PANSS scores began to decrease (mean ΔPANSS_ECT1_ was 11.7%; Range, 2%-32.8%), decreased significantly after the eighth application (mean ΔPANSS_ECT8_ was 86.0%; Range,72.5% to 97.9%). Five patients met the response criteria after ECT1 (20% reduction in PANSS total score), and all patients met the response criteria after ECT8. (3) Linear regression analysis showed that ΔPANSS_ECT1_ was a significant predictor of ΔPANSS_ECT8_ (*F*=5.387, *P*=0.028), and ΔPANSS_ECT1_ explained 15.7% of the variance of ΔPANSS_ECT8_ (*R*^*2*^=0.157).

**Conclusions:**

ECT was able to normalize γ, λ, σ, Ne of INS_R_vIa, NLe of bilateral FuG_A37mv in SZ patients after the first treatment, and NLe of MVOcC_R_rLinG after the eighth ECT. ECT significantly alleviates psychotic symptoms in patients with SZ, and its efficacy after eight sessions can be predicted by the patient's response to the first session of ECT.

**Supplementary Information:**

The online version contains supplementary material available at 10.1186/s12888-023-05408-1.

## Background

Schizophrenia (SZ) is a disabling severe psychiatric disorder with positive symptoms (delusions, hallucinations, and formal thought disorders) and negative symptoms (lack of volition, reduced verbal output, and reduced emotional expression) as the main clinical manifestations, with a lifetime prevalence of 1% [1], the suicide rate is about 5% [2], causing a huge burden on the individual and social life. However, the etiology of schizophrenia is unknown, and the clinical diagnosis and treatment of schizophrenia are mainly based on symptoms, and there is a lack of objective biological diagnostic criteria. Clinical treatment is currently dominated by medication, but up to 30% of schizophrenia patients do not respond or respond poorly to standard antipsychotic medication [3].

Electroconvulsive therapy (ECT) can shorten the duration of positive symptoms and increase the rate of remission in patients who do not respond well to medication [4]. By briefly inducing general anesthesia, ECT can transfer an appropriate amount of electric charge to the brain through scalp electrodes to cause generalized seizures lasting 20-60 seconds, so as to relieve symptoms [5]. However, the exact mechanism of treatment is unclear and different previous studies have shown different remission rates [6–8]. Further studies are necessary to further elucidate the potential therapeutic mechanisms of ECT and to identify patients who may ultimately achieve the greatest therapeutic benefit.

Numerous neuroimaging studies have shown that schizophrenia does not merely result from injury of one or a few independent brain regions; Rather, it may be the product of pathological alterations distributed throughout a complex interconnected brain network [9]. Complex brain network analysis aims to use some indicators with neurobiological significance to characterize the brain network based on different imaging methods, such as quantitative analysis of brain area connectivity in functional magnetic resonance imaging (fMRI). fMRI uses blood oxygen level-dependent signals as the basis for imaging and non-invasive visualization to infer functional brain activity. It has the advantages of no ionizing radiation, good reproducibility, and high spatial resolution [10]. There have been previous studies on MRI-based ALFF, FC [11] and gray matter volume [12] methods to explore the changes of schizophrenic patients before and after ECT, but there is no study on graph theory methods. The graph theory is the tool used to characterize the network , which describes the brain network based on the effective degree of information exchange [13]. It reflects the efficiency of information transfer in brain networks, including both global and nodal properties. In this study, the graph theory approach was adopted, in which global properties include global efficiency (Eglo) that measure the ability of a network to transmit information simultaneously, local efficiency (Eloc) that measure the fault tolerance of the network, which represents the communication between the neighbors of the node after a node is deleted, clustering coefficient (Cp) that measure the stability of local connections between network nodes and the ability of the network to resist external interference, normalized clustering coefficient (γ) that represents the ratio of the clustering coefficient of the real network to 100 random networks, shortest path length (Lp) that measure the path length of two nodes in a network with the least number of edges, normalized shortest path length (λ) that represents the ratio of the shortest path length of the real network to 100 random networks, small-word network (σ) that has a relatively small λ and large γ, and node properties including Ne and NLe. Previous studies have found that Ne of the right hypooccipital cortex [14] and NLe of basal ganglia [15] were lower in patients with schizophrenia than in healthy controls, but there is no graph theory exploration to explore the mechanism of ECT therapy in schizophrenia. In this study, we analyzed the differences in these attribute values in schizophrenic patients before and after ECT and compared with healthy controls on the basis of fMRI to explore brain functional network patterns in schizophrenic patients.

According to previous studies [16] and our clinical observations, patients' symptoms can be improved even during the initial phase of ECT application (including after a single ECT session). Based on this, the present study hypothesized that changes in brain network patterns may occur in schizophrenia patients with ECT, and that these changes in brain network patterns may be associated with changes in patients' clinical symptoms. The aim of this study was to investigate the changes of brain functional network in patients with schizophrenia induced by ECT by acquiring clinical assessments and fMRI data before and after first ECT, and after eight ECT treatment in schizophrenia patients.

## Methods

### Study design

All patients who met the inclusion criteria had general information, clinical assessment and MRI scans collected before the first ECT (Pre-ECT), received clinical assessment and MRI scans within 10-12 hours after the first ECT (ECT1) and the eighth ECT (ECT8). Additionally, general information and MRI scan data of healthy controls were collected. Clinical symptoms were assessed using the Positive and Negative Syndrome Scale (PANSS), which includes three factors: positive symptoms, negative symptoms and general psychopathology. Patients were defined as responders when there was a decrease of ≥20% in PANSS total score [17]. The relevant definition is as follows:

$${\Delta PANNSS}_{ECTt}=\left|\frac{{\mathrm{PANNS}}_{\mathrm{Pre}-\mathrm{ECT}}-{\mathrm{PANNSS}}_{\mathrm{ECTt}}}{{\mathrm{PANNS}}_{\mathrm{Pre}-\mathrm{ECT}}-30}\right|\times 100\%$$

ΔPANSS_ECTt_ represents how much the total PANSS score decreased after t ECT, PANSS_Pre-ECT_ represents the patient's total PANSS score before ECT, PANSS_ECTt_ is the total PANSS score after t ECT, and 30 is the "asymptom" score of the total PANSS score.

### Participants

Thirty-one patients with schizophrenia who were hospitalized in the Second Affiliated Hospital of Xinxiang Medical University and scheduled to undergo ECT were included in this study. Inclusion criteria: (1) had been given a medical prescription for ECT; (2) diagnosed with schizophrenia by two or more attending physicians through a structured clinical interview on DSM-IV; (3) PANSS total score ≥ 60; (4) bout 18~50 years old; right-handed; (5) had not received ECT before. Exclusion criteria: (1) comorbid other psychiatric disorders (2) had drug or alcohol dependence; (3) suffering from severe physical diseases such as cardiac, hepatic, or renal insufficiency; (4) patients with contraindications to MRI (metal implants in the body, claustrophobia, etc.); (5) history of major cranial trauma (loss of consciousness for >5 min).

Fifty healthy subjects who were well matched to the patients in terms of age, gender, and years of education were recruited during the same period. Exclusion criteria included a history of mental illness in the individual or his family, and the rest exclusion criteria as in the patient group.

This study has been approved by the Ethics Committee of the Second Affiliated Hospital of Xinxiang Medical University (ethical approval number: XYEFYLL-(Research)-2021-33). All participants signed the informed consent form.

### Treatment

ECT treatment: The course of ECT was 8 times, 3 times a week. Before ECT treatment, electrocardiogram, chest X-ray, blood routine and blood biochemistry were checked, weight was measured, routine water and food fasting for 6 hours, and intravenous general anesthesia was used. Atropine 0.01mg/kg, propofol 2mg/kg, succinylcholine chloride 1mg/kg were injected into the vein, and Thymatorn TM DGx IV modified electroconvulsive therapy device from American Eagle Medical Technology was used to treat the patient, and the electrodes were placed on bitemporal placement. The parameters of ECT were as follows: output current = 0.9 A, maximum charge = 504 mc, frequency = 10-70 Hz, pulse width = 1.0 ms, maximum stimulation duration = 8 s. The antipsychotic drug regimen was kept constant throughout the treatment, including the type, dosage and duration of drug administration.

### Clinical symptom assessment

The presence and the severity of each clinical symptom in patients with schizophrenia were assessed using the PANSS scale. The scale consisted of 30 items , including 7 positive items (P1-P7), 7 negative items (N1-N7), and 16 general psychopathology items (G1-G16).

### Magnetic resonance data acquisition and preprocessing

MRI data were acquired by a Siemens 3.0T superconducting MRI (MAGNETOM Verio, Germany) imaging system at the Radiology Department of the Second Affiliated Hospital of Xinxiang Medical University. The head was placed in the center of the coil and the gap between the coil and the head was fixed with a matching foam pad to reduce motion artifacts, and the ears were covered with special earphones and rubber earplugs to reduce noise interference. The subjects were asked to lie supine, close their eyes, stay awake and unable to fall asleep, and try not to engage in any mental activity during the whole scanning process. Conventional T1 and T2 weighted imaging scans were used to exclude organic lesions and structural abnormalities of the head, and then gradient echo-echo planar imaging (GRE-EPI) sequence was used to acquire functional data. The specific parameters were: repetition time (TR) = 2000 ms, echo time (TE) = 30 ms, layer thickness/layer spacing = 4.0 mm/0.8 mm, field of vision = 220 × 220 mm^2^, matrix = 64 × 64, and flip angle (FA) = 90°.

The acquired images were preprocessed by the Data Processing Assistant for Resting-State fMRI (DPARSF 2.3. http://www.restfmrrestfmri.t/forum/DPARnet/forum/DPARSFV2.3.) on the Matlab 2013b platform. The steps are as follows: (1) the original DICOM format is converted to NIFTI format; (2) In order to eliminate the influence of the initial machine signal instability, the uneven magnetic field, the subject's unadaptability to the test environment, and the instrument noise on the results, the first 10 time points were deleted, and the 230 time points of the resting state were retained. (3) Time correction was performed by taking the middle layer (layer 33) as reference; (4) Head movement correction: subjects whose head movement was more than 2mm in translation and more than 2° in rotation were excluded. (5) Spatial normalization: Firstly, the functional image was registered with the T1 structural image, and then the T1 structural image was registered to the Montreal Neuroscience Institute (MNI) standard space by DARTEL separated registration method. (6) The image was smoothed with a Gaussian kernel of 6mm×6mm×6mm FWHM to improve the image signal-to-noise ratio and make the image closer to the random Gaussian field model. (7) Removing confounding signals by regression analysis of 24 head motion parameters, mean white matter signal and cerebrospinal fluid signal as covariates. (8) Filter detrend in the range of 0.01-0.08HZ to eliminate the covariate effects of low-frequency linear displacement and high-frequency noise caused by respiration and heartbeat.

### Construction of THE functional network

Network nodes and edges were constructed using GRETNA 2.0 (https://www.nitrc.org/Projects/gretna). (1) Define brain network nodes: the whole brain was divided into 246 brain subregions using Brainnetome Atlas, which includes 210 cortical regions and 36 subcortical regions, and each subregion represents a network node; (2) Define brain network edges: the mean time series of each node was extracted, and the mean time series between all node pairs was calculated as Pearson correlation as brain connections (edges). This will result in a 246 × 246 correlation matrix for each participant; (3) To reduce the possibility of false positive connections for all subjects, these individual correlation matrices are binarized within a certain connectivity density threshold to obtain a set of binary networks. In this study, the sparsity threshold ranges from 0.05 to 0.50 with an increment of 0.05, which is defined as the ratio of the available number of edges in the network to the maximum number of possible edges in all correlation matrices.

### Brain network topological properties analysis

GRETNA2.0 software was used to calculate the area under the curve (AUC) of the topological properties of nodes in the whole brain network in all threshold ranges, and these AUC values were used for statistical comparison. (1) Eglob: It is a global measure of the parallel information transfer (communication) capability of the entire network. It is calculated as the average of the inverse of the "summed average" of the characteristic path lengths. $Eglob=\frac{1}{N\left(N-1\right)}\sum_{i\ne j\in G}\frac{1}{{L}_{\mathrm{i}j}}$ where N is the number of nodes in the graph G,${L}_{ij}$ denotes the shortest path length between nodes i and j; (2) Eloc: It measures the fault tolerance of the network, indicating the information communication between the neighbors of a given node when the node is deleted:$Eloc=\frac{1}{N}\sum_{i\in G}Eglob\left({G}_{i}\right)$ , where Gi is a subgraph consisting of the nearest neighbors of node i and the connections between them; (3) Cp: It portrays the stability of local connections between network nodes and the ability of the network to resist external disturbances:. $Cp=\frac{1}{N}\sum_{i\subseteq N}\frac{{\sum }_{j,\mathrm{h}\subseteq N}{\left({a}_{ij}{a}_{i\mathrm{h}}{a}_{j\mathrm{h}}\right)}^\frac{1}{3}}{{k}_{i}\left({k}_{i}-1\right)}$ , a_ij_ , a_ih_ , and a_jh_ are the connection values between nodes i, j and h in the functional connectivity matrix A, respectively. N is the set of all nodes in the functional connectivity matrix, and Ki denotes the degree of node i, that is, the weight sum of the functional connectivity values between node i and other network nodes; (4) Lp: the minimum number of edges connecting two nodes in the network is the path length of those two points, and the average of the path lengths of all node pairs in the network is the characteristic path length of the network. ${L}_{P}=\frac{1}{N\left(N-1\right)}\sum_{i\in G}\sum_{j\in G}\frac{1}{{L}_{ij}}$ ; N is the number of nodes in the graph G, and ${L}_{ij}$ is the shortest path length between nodes i and j. (5) γ: the ratio of the clustering coefficient of the real network to the clustering coefficient of the 100 random networks: γ = Creal/Crandom, Crandom is the clustering coefficient of the random network and Creal is the clustering coefficient of the real network. (6) λ: the ratio of the characteristic path length of the real network to the characteristic path length of the 100 random networks: λ= Lreal/Lrandom≈1, Lrandom is the shortest path length of the random network, Lreal is the shortest path length of the real network. (7) σ: It refers to its relatively small characteristic path length and relatively large clustering coefficient, that is, the connections between local brain regions are dense, and at the same time, any two brain regions communicate quickly with each other through a small number of connections, calculated as σ = γ / λ. When σ > 1, γ > 1 and λ ≈ 1, the network is considered to have the small-world property. (8) Ne: the reciprocal of the average of the sum of the shortest path lengths between this node and all other nodes. $N\mathrm{e}\left(i\right)=\frac{1}{N-1}\sum_{i\ne j\in G}\frac{1}{{L}_{ij}}$ ; G is the set of all nodes in the network. ${L}_{ij}$ denotes the shortest path length between nodes i,j; (9) NLe: the information communication efficiency between the neighbors of this node when this node is removed. $NL\mathrm{e}\left(i\right)=\frac{1}{{N}_{{G}_{i}}\left({N}_{{G}_{i}}-1\right)}\sum_{j\ne k\in G}\frac{1}{{L}_{jk}}$ ; G_i_ refers to the subgraph formed by the neighbors of node i, and L_jk_ denotes the shortest path length between nodes j, k.

### Statistical analysis

Independent samples t-test was used to compare whether there were statistical differences in age and years of education between the SZ and healthy control group, χ^2^ was used to compare whether there were statistical differences in gender between the two groups. Brain network topological properties values were extracted, then independent sample t-tests were used to compare differences in brain network global (*p* < 0.05, FDR-corrected) and nodal (*p* < 0.0002, Bonferroni-corrected) topological properties values between the SZ and HC groups with age, gender, and years of education as covariates. Differences in brain network global (*p* < 0.05, FDR-corrected) and nodal (*p* < 0.0002, Bonferroni-corrected) topological properties values among Pre-ECT, ECT1, and ECT8 were assessed using repeated measures ANOVA, and then post hoc analyses were used to derive exactly which two groups were different from each other. The correlation between changes of brain network properties values that differed between groups and changes in clinical symptom scores was assessed using partial correlation analysis with age, gender, years of education, and duration of illness as covariates, and FDR correction was performed. Linear regression analysis was used to explore the relationship between the first and eighth treatment responses in the SZ group.

## Results

### Demographic and clinical characteristics

A total of 31 patients with SZ and 50 healthy controls were finally enrolled. There were no statistically significant differences between groups in gender composition ratio (X^2^ =0.005), age (t=0.136), and years of education (t=-1.602). The descriptive demographic and clinical characteristics of the participants are shown in Table 1.

**Table 1:** Demographic and clinical characteristics of patients and HCs

Variables	Patients		HC		*P*
	Pre-ECT	ECT1	ECT8		
Gender(male/female)^a^	17/14		27/23		0.941
Age, y^b^	30.23±7.873		29.96±8.775		0.892
Education, y^b^	11.77±3.783		12.98±2.952		0.113
Illness duration, mo	10.94±8.96		-		-
PANSS				-	-
Positive	20.35±4.10	19.03±4.52	8.97±1.56	-	-
Negative	20.68±3.16	20.55±3.16	8.87±1.69	-	-
General	36.55±3.72	34.68±3.73	18.71±1.51	-	-
Total	78.48±6.01	75.10±5.76	36.94±3.28	-	-
Medication doses, mg/d	441.19 ±82.61.00			-	-

^a^Two-tailed χ2 test

^b^Two-tailed t tests; Medication doses: doses calculated from power transformation for chlorpromazine equivalent

### Comparison 1: pre-ECT vs HC

**Fig. 1:**
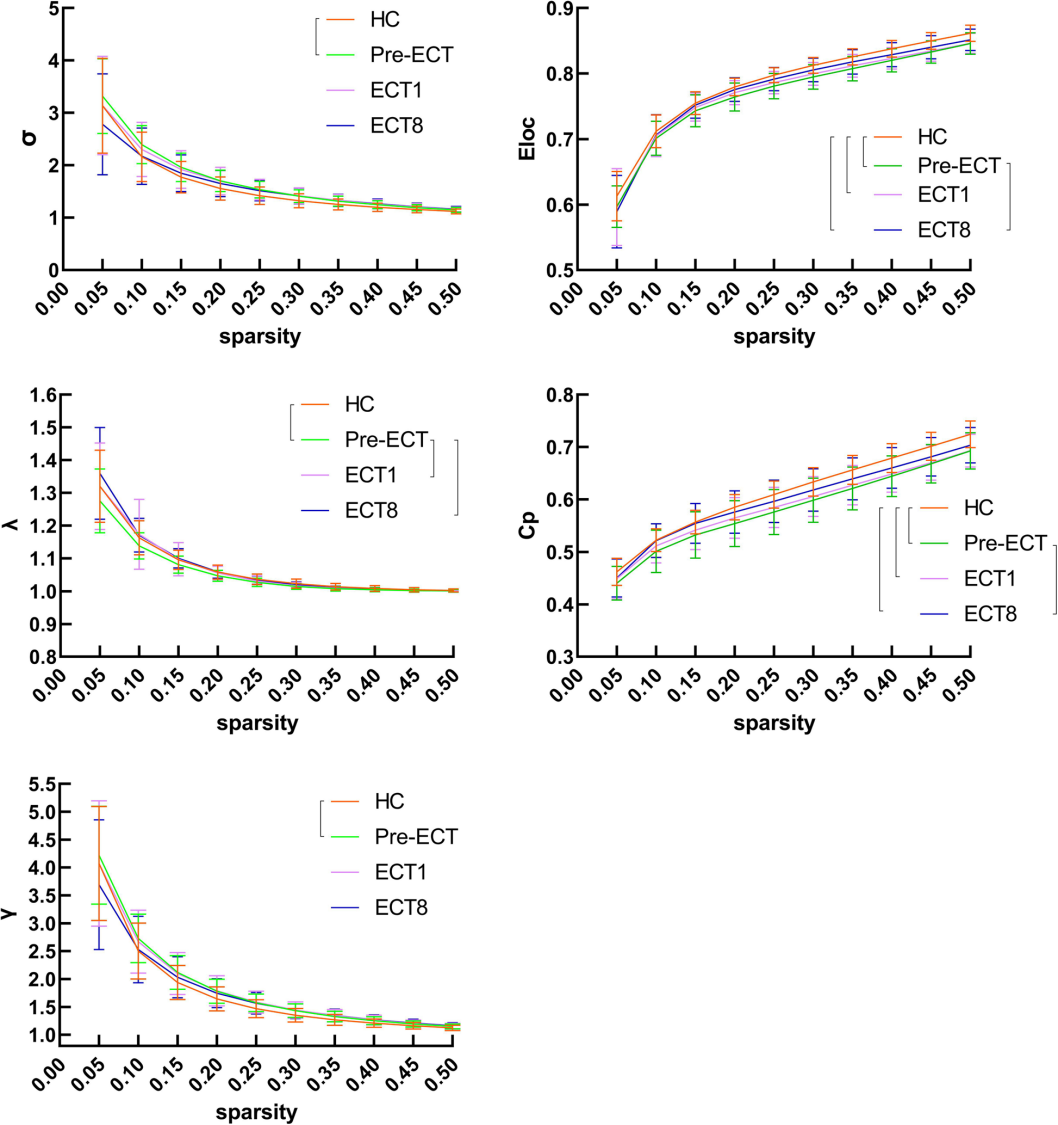
Broken line diagram of progressive changes of brain network global measures induced by ECT. The t test analysis was conducted among groups (*p*< 0.05, FDR corrected). HC, Healthy control group; Pre-ECT, before the ECT; ECT1, after the first ECT; ECT8, after the eighth ECT; Eloc, local efficiency; Cp, clustering coefficient; γ, normalized clustering coefficients; λ, normalized shortest path length; σ, small-word network. [ symbol indicate statistical differences between these two groups

**Fig. 2:**
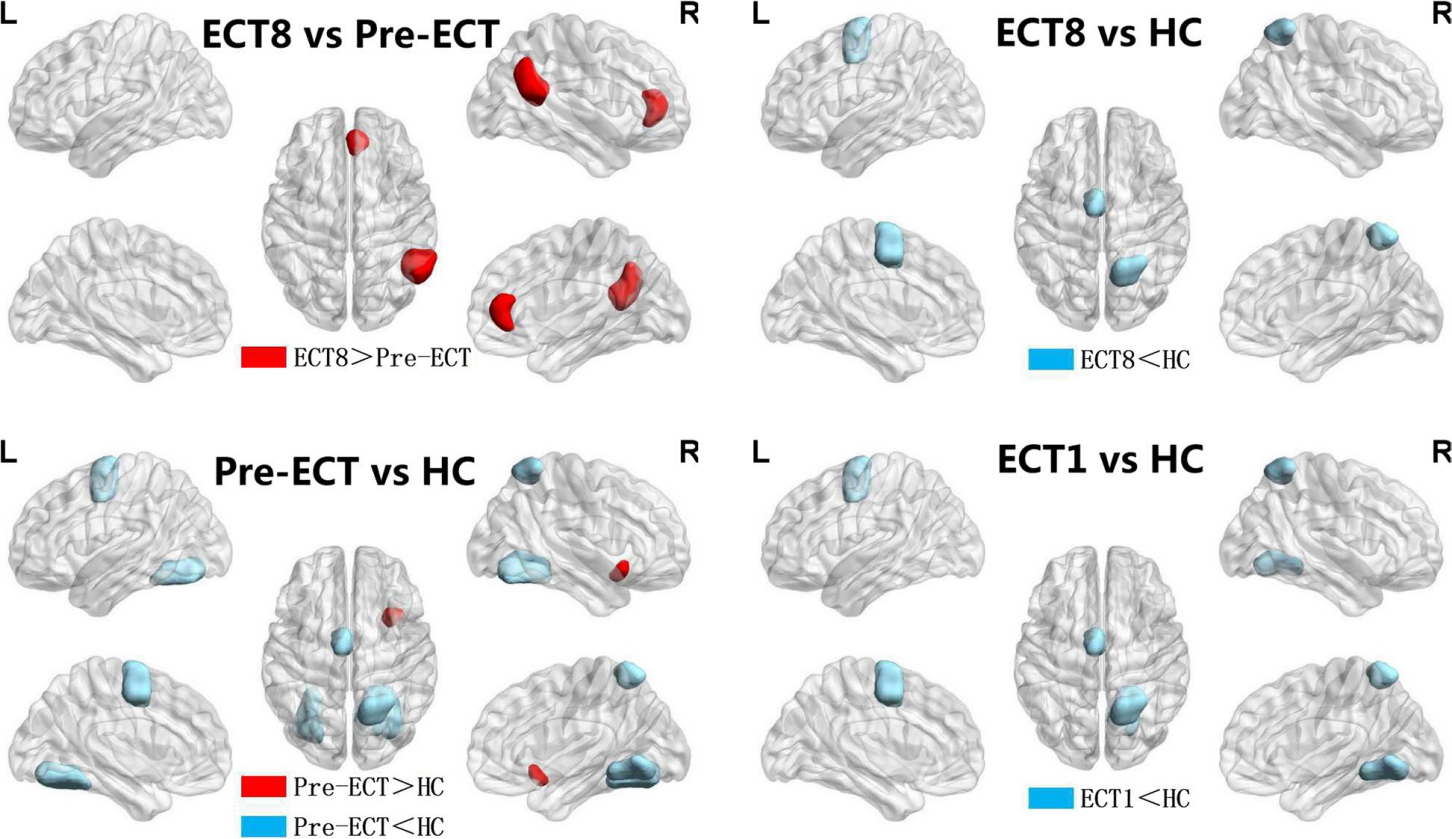
Images of progressive changes of brain network nodal measures induced by ECT. The *t* test analysis was conducted between groups (*p* < 0.0002, Bonferroni corrected). HC, Healthy control group; Pre-ECT, before the first ECT; ECT1, after the first ECT; ECT8, after the eighth ECT; L, left; R, right. The color blocks in the figure represent different brain regions

Compared with the HC group, the SZ group showed decreased Eloc, Cp and λ values, increased γ and σ values; Ne values of right insular gyrus ventral agranular insula (INS_R_vIa) were increased, SPL_R_A7r were decreased. NLe values of bilateral FuG_A37mv, right medio ventral occipital MVOcC _R_rLinG, SFG_L_A6m were decreased. Patients' γ and σ values were positively correlated with negative PANSS scores (*r* = 0.415,0.436; *P* =0.031, 0.023), total PANSS scores (*r* = 0.419, 0.436; *P* = 0.029, 0.023); Ne values of SPL_R_A7r were negatively correlated with negative PANSS scores (*r* = -0.589, *P* = 0.001) (Tables 2 and 3; Figs. 1 and 2).

**Table 2:** Comparison of brain network topological properties between Patients and HCs groups

property (brain subregion)	Anatomical and modified Cyto-architectonic descriptions	HC	Pre-ECT	ECT1	ECT8	Pre-ECT vs HC	ECT1 vs HC	ECT8 vs HC
		($\overline{x }$ ±s)				(t value)		
**Eloc**		0.36±0.00	0.35±0.01	0.35±0.01	0.35±0.01	↓(-5.00)	↓(-4.19)	↓(-2.69)
**Cp**		0.28±0.01	0.26±0.02	0.27±0.01	0.27±0.02	↓(-4.64)	↓(-3.94)	↓(-2.02)
** γ**		0.76±0.10	0.8±0.09	0.80±0.11	0.78±0.11	↑(2.20)	→	→
** λ**		0.48±0.01	0.47±0.01	0.48±0.01	0.48±0.01	↓(-2.56)	→	→
** σ**		0.70±0.09	0.75±0.08	0.74±0.10	0.72±0.10	↑(2.52)	→	→
**Ne**								
INS_R	ventral agranular insula	0.23±0.03	0.264±0.02	0.255±0.02	0.249±0.03	↑(4.50)	→	→
SPL_R	rostral area 7	0.27±0.03	0.22±0.05	0.24±0.04	0.23±0.04	↓(-5.17)	↓(-4.92)	↓(-5.35)
**NLe**								
FuG_L	medioventral area37	0.37±0.02	0.33±0.04	0.35±0.03	0.34±0.05	↓(-4.87)	→	→
FuG_R	medioventral area37	0.37±0.02	0.34±0.03	0.35±0.03	0.35±0.04	↓(-4.53)	→	→
MVOcC_R	rostral lingual gyrus	0.38±0.02	0.35±0.02	0.36±0.02	0.36±0.02	↓(-5.75)	↓(-3.96)	→
SFG_L	medial area	0.36±0.02	0.35±0.02	0.34±0.02	0.35±0.02	↓(-4.00)	↓(-4.31)	↓(-4.00)

↓ represents decreased values of brain network topological properties in the patient group than in the HCs group

↑ represents increased values of brain network topological properties in the patient group than in the HCs group

→ represents no statistical difference in the values of brain network topological properties between the patient group and the HCs group

**Table 3:** Correlation between brain network topology attribute values and PANSS scores in patients with schizophrenia

	Positive PANSS scores		Negative PANSS scores		Total PANSS scores	
	r	*P*	r	*P*	r	*P*
**Pre-ECT**						
γ	-	-	0.415	0.031	0.419	0.029
σ	-	-	0.436	0.023	0.436	0.023
Ne of SPL_R_A7r	-	-	-0.589	0.001	-	-
**ECT1**						
Ne of SPL_R_A7r	-0.408	0.035	-	-	-	-
NLe of MVOcC_R_rLinG	-	-	0.462	0.007	-	-

*Pre-ECT* Before the first ECT, *ECT1* After the first ECT, *SPL_R_A7r* Right superior parietal lobule rostral area 7, *MVOcC_R_rLinG* Right medio ventral occipital cortex rostral lingual gyrus, *γ* Normalized clustering coefficient, *σ* Small-word network, *Ne* Nodal global efficiency, *NLe* Nodal local efficiency

### Comparison 2: ECT1 vs HC

Compared with the HC group, the SZ group showed decreased Eloc and Cp values; decreased Ne values of SPL_R_A7r and NLe values of MVOcC_R_rLinG, SFG_L_A6m. Patients' global properties values were not statistically significant in correlation with clinical symptom score values; Ne values of SPL_R_A7r were negatively correlated with positive PANSS scores (*r*=-0.408, *P*=0.035), NLe values of MVOcC _R_rLinG were positively correlated with negative PANSS scores (*r*=0.462, *P*=0.007) (Tables 2 and 3; Figs. 1 and 2).

### Comparison 3: ECT8 vs HC

Compared with the HC group, the SZ group showed decreased Eloc and Cp values; decreased Ne values of SPL_R_A7r and NLe values of SFG_L_A6. Patients' global and nodal properties values were not statistically significant in correlation with clinical symptom score values (Table 2; Figs. 1 and 2).

### Comparison 4: ECT1 vs Pre-ECT

Compared with the Pre-ECT, Patients showed increased λ values after the first ECT application. Patients' Δλ (λ (ECT1) − λ (Pre-ECT)) values were negatively correlated with negative ΔPANSS ECT1 scores (*r*=-0.444. *P*=0.020) (Tables 4 and 5; Fig. 1).

**Table 4:** Comparison of brain network topological properties before and after ECT in the Patients group ($\overline{x }$ ±s)

property(Brain subregion)	Anatomical and modified Cyto-architectonic descriptions	Pre-ECT	ECT1	ECT8	ECT1 vs Pre-ECT	ECT8 vs Pre-ECT
Eloc		0.35±0.01	0.35±0.01	0.35±0.01	→	↑(2.42)
Cp		0.26±0.02	0.27±0.01	0.27±0.02	→	↑(2.46)
λ		0.47±0.01	0.48±0.01	0.48±0.01	↑(2.137)	↑(3.11)
NLe						
IPL_R	rostroventral area 39	0.35±0.02	0.36±0.02	0.37±0.02	→	↑(5.34)
CG_R	subgenual area 32	0.35±0.02	0.36±0.02	0.37±0.02	→	↑(4.27)

↑ represents increased values of brain network topological properties in the patient group after treatment than before treatment.

→ represents no statistical difference in brain network topological propertie values before and after treatment in the patient group.

**Table 5:** Correlation between changes in brain network topology attributes and changes in PANSS scores in schizophrenia during ECT

	△Negative PANSS scores		△Total PANSS scores	
	r	*P*	r	*P*
**ECT1-Pre-ECT**				
△λ	-0.444	0.020	-	-
**ECT8-Pre-ECT**				
△Cp	-	-	-0.405	0.036

*Pre-ECT* Before the first ECT, *ECT1* After the first ECT, *ECT8* After the eighth ECT, λ Normalized shortest path length; *Cp* Clustering coefficient

### Comparison 5: ECT8 vs Pre-ECT

Compared with the Pre-ECT, Patients showed increased Eloc, Cp and λ values; increased NLe values of right Inferior Parietal Lobule rostroventral area 39 (IPL_R_A39rv), Cingulate Gyrus subgenual area 32 (CG_R_A32sg) after the eighth ECT application. Patients' ΔCp (Cp (ECT8) − Cp (Pre-ECT)) values were negatively correlated with the total ΔPANSS scores (*r*=-0.405, *P*=0.036) (Tables 4 and 5; Figs. 1 and 2).

### Comparison 6: ECT8 vs ECT1

There were no differences in all properties of patients after the eighth ECT application compared to after the first ECT application.

### Predictive efficacy of ECT1 response

**Fig. 3:**
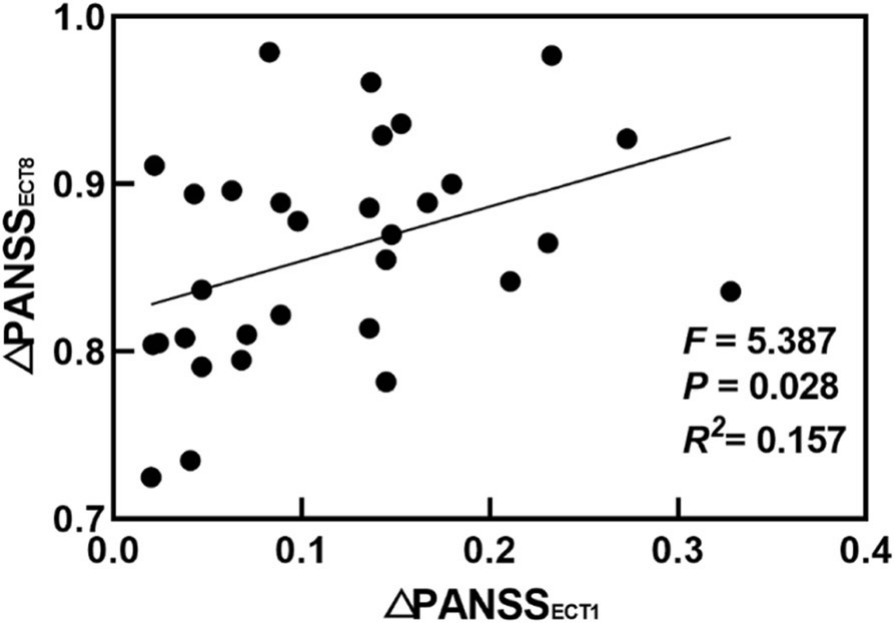
Linear regression analysis between the ΔPANSS_ECT1_ and ΔPANSS_ECT8_. ΔPANSS_ECT1_ presents the reduction rate of PANSS total scores in patients after the first ECT. ΔPANSS_ECT8_ presents the reduction rate of PANSS total scores in patients after the eighth ECT

Even after only the first application of ECT, the total PANSS score had begun to decrease (mean ΔPANSS_ECT1_ of 11.7%; range, 2% ~32.8%) and decreased significantly after the eighth application (mean ΔPANSS_ECT8_ of 86.0%; range, 72.5%-97.9%). Five patients met the response criterion (20% reduction in total PANSS score) after ECT1 and all patients met this response criterion after ECT8. Linear regression analysis showed that ΔPANSS_ECT1_ was a significant predictor of ΔPANSS_ECT8_ (F = 5.387, *p* = 0.028), and ΔPANSS_ECT1_ explained 15.7% of the variance of ΔPANSSECT8 (*R*^2^ = 0.157) (Fig. 3).

## Discussion

The aim of this study was to investigate the progressive changes in the topological properties of the brain functional network during multiple consecutive ECT treatments in patients with SZ. Functional brain imaging techniques and Brainnetome Atlas were used to construct brain networks. The magnetic resonance imaging information of the patients' brain was acquired before ECT, after first and eighth ECT respectively, and then the process of changes in the information transfer efficiency of the patients' brain functional networks was analyzed. And the changes of functional connectivity of these brain networks were related to the improvement of clinical symptoms.

ECT has a significant effect on improving psychiatric symptoms in SZ [18]. Usta et al [6] found that 95% of patients in the ECT group responded to treatment (total PANSS score reduction of at least 25%). The results of a retrospective study showed that 81% of patients with SZ had a decrease in total PANSS scores of more than 20% [8]. A meta-analysis involving 22 randomized controlled studies showed that the combination of antipsychotics and ECT met study-specific criteria for "clinical improvement" at a much higher rate than treatment with antipsychotics alone [7]. In this study, it was found that the average reduction rate of total PANSS in SZ patients was 11.7% after ECT1 and 86% after ECT8 (ranging from 72.5% to 97.9%, and all patients met the 20% reduction in PANSS total score), further confirming that the combination application of ECT and antipsychotic medication is effective in relieving clinical symptoms in SZ. Quick treatment effect is one of the key advantages of ECT [19]. Studies found that a single ECT can have a significant impact on the patient's symptoms [16] and brain structure [20]. In the present study, we not only found that patients' clinical symptoms were relieved after ECT1, but also found that the efficacy of the first ECT application predicted the efficacy of the eventual eighth ECT application (i.e. the more symptom reduction after ECT1, the better symptom recovery after ECT8). These findings could help clinicians focus earlier on which patients are likely to end up better treated.

It is believed that ECT can restore brain function connectivity in patients with psychiatric disorders [21], and promote neurological recovery in patients' pathological brain regions. In this study, two-by-two comparisons of the values of brain network topological properties of patients Pre-ECT, ECT1 and ECT8 with HC were made, and it was found that small-world properties of brain network topology existed in both the patient group and healthy controls, i.e., σ > 1, γ > 1, and λ ≈ 1. However, σ was increased in the patient group than in the healthy controls before ECT. The application of ECT1 alone could restore the SZ patients' γ, λ σ, Ne of INS_R_vIa, NLe of bilateral FuG_A37mv back to normal, NLe values of MVOcC_R_rLinG, although still decreased than HC after ECT1 treatment, eventually returned to normal after ECT8 as well. Previous studies have shown increased γ values in the cortical structural network in the SZ patient group compared to healthy controls [22]. Jia S et al. [23] constructed networks by EEG and found that SZ patients had decreased characteristic path lengths. Functional neuroimaging studies also showed a significant increase in σ in whole brain connectivities in schizophrenia patients in a working memory (WM) task [24]. This is consistent with the results of the present study, which both confirm the abnormal σ in SZ patients, indicating an imbalance between local information transmission throughout the brain and global network efficiency, but in the present study normalized after a single ECT application. Ventral insula belongs to the ventral attention network [25] that is involved in emotion processing [26], and abnormal feedback of information from external stimuli is associated with abnormalities in the structure and function of the insula in SZ patients [27]. Consistent with the results of the present study, Sun-Young Moon [28] also pointed to the insula as other potential neural substrates that may be disease-specific alterations in ECT-induced schizophrenia. The pallidum (bilateral FuG_A37mv) is a very important core brain region for visual processing of the face and cognitive processing of logical information in SZ patients [29]. Abnormalities in the nodal efficiency of the syrinx gyrus have also been identified in previous studies [14]. The occipital cortex may be the neurobiological basis for early visual processing deficits in schizophrenia [30]. A functional imaging study found low nodal efficiency in the occipital region of SZ patients by constructing dynamic brain networks [23]. The study results of Hu H et al [26] pointed out that the brain network connections in the right inferior occipital gyrus were altered in SZ patients after treatment compared with those before ECT treatment. This study also found that the NLe value of MVOcC_R_rLinG in SZ patients after ECT1 was positively correlated with negative PANSS scores.

A pairwise comparison of SZ patients before and after ECT treatment revealed that λ was elevated in ECT1 compared to Pre-ECT and was increased after ECT8. This corroborates with the finding in this study that λ was decreased in SZ patients at baseline compared to the HC group and improved over after a single ECT application. ECT8 showed elevated NLe of both IPL_R_A39rv and CG_R_A32sg compared to Pre-ECT, and the change in Cp value was negatively correlated with the rate of subtraction from the PANSS scale total score. Dysfunction in the inferior parietal lobule is thought to be responsible for working memory deficits in SZ patients, and working memory deficits have been considered to be central to SZ-related cognitive deficits [31]. Clinical research evidence suggests increased functional connectivity between the inferior parietal lobule and visual and sensorimotor areas in patients with SZ [32]. Liu F et al [33] demonstrated for the first time that a disruption of topological properties was demonstrated in SZ patients through a cerebral blood flow covariance network, which included abnormalities in the nodal centrality of the inferior parietal lobule. The cingulate gyrus is the core brain region of the default network [34]. It plays a role in executive functions such as learning, processing and memory [35]. One researcher have found that the cingulate cortex in the SZ patient group exhibited decreased nodal local efficiency compared to healthy controls [36]. Based on this, the present study also found that ECT application resulted in increased local efficiency of cingulate nodes in SZ patients, suggesting that ECT may be used to treat patients' clinical symptoms by modulating their brain network functional connectivity.

Both Eloc and Cp were elevated in ECT8 compared to Pre-ECT, and the change in Cp value was negatively correlated with the rate of reduction in PANSS scale total score. eloc and Cp, although consistently different from the HC group after both ECT1 and ECT8, were shown by t-values to decrease gradually with treatment. hu H et al [14] found a trend toward decreased Eloc in the SZ group compared with the HC group at baseline; Eloc was elevated in the fMRI network of the SZ group after treatment compared with that before ECT treatment. A study using dynamic time-varying brain functional connectivity based on a graph-theoretic approach found that SZ patients showed decreased clustering coefficients in all temporal states [37]. This is consistent with the results of the present study, both suggesting that SZ patients have less tolerant brain networks, less aggregation between neighboring brain regions, and reduced local information exchange. Ne of SPL_R_A7r and NLe of SFG_L_A6m also consistently differed from the HC group before and after ECT application, but showed no signs of improvement. The superior parietal lobule is a functionally diverse region involved in many functions such as visuospatial and visuomotor integration, attentional processing, memory and language tasks [38]. It is probably because of the multiple functions involved that some clinical symptoms remain unimproved in SZ patients after ECT, so that SPL_R_A7r abnormalities are consistently present before and after ECT. The superior frontal gyrus (SFG) is involved in many cognitive and motor control tasks [39]. Previous studies have also pointed out that patients with SZ exhibit decreased nodal local efficiency in the superior frontal gyrus [36]. It is known that ECT can cause some cognitive side effects [40]. This may explain the difference in nodal local efficiency of the superior frontal gyrus between SZ patients and healthy controls even after ECT.

There are several limitations of the present study. First, the present study only initially elucidated brain network connectivity abnormalities in SZ patients from a functional perspective, and did not investigate them in conjunction with structural connectivity, for example. Future studies combining different methods on a multimodal basis are needed. Second, this study only measured the severity of patients' clinical symptoms and lacked the assessment of patients' cognitive ability, and should use a set of cognitive assessment tools to tap into patients' multidimensional cognitive information. Third, the long-term effects of ECT on SZ patients were not tracked longitudinally. Further longitudinal studies are needed in the future to track patients' ECT treatment effects over time. Fourth, PANSS also has some limitations of the disorders of formal thinking are not optimally mapped with this.

## Conclusions

In this study, based on fMRI, the graph theory method was applied to study the influence of ECT on the brain functional network connectivity of SZ patients, and the improvement of ECT on the clinical symptoms of SZ patients was evaluated by PANSS scale. It was found that ECT normalized γ, λ, σ, Ne of INS_R_vIa, and NLe of bilateral FuG_A37mv in SZ patients after the first treatment, and NLe of MVOcC_R_rLinG after the eighth ECT. ECT significantly alleviates psychotic symptoms in patients with SZ, and its efficacy after eight sessions can be predicted by the patient's response to the first ECT. ECT may improve clinical symptoms by regulating the connectivity of brain functional network in patients with SZ.

### Supplementary Information

**Additional file 1.** 

## Data Availability

The datasets and materials generated or analyzed during the current study are not publicly available due to confidentiality but are available from the corresponding author on reasonable request.
